# Social meanings and understandings in patient-nurse interaction in the community practice setting: a grounded theory study

**DOI:** 10.1186/1472-6955-11-14

**Published:** 2012-09-05

**Authors:** Kathleen M Stoddart

**Affiliations:** 1 School of Nursing, Midwifery and Health, University of Stirling, Stirling, FK9 4LA, Scotland

## Abstract

**Background:**

The patient-nurse relationship is a traditional concern of healthcare research. However, patient-nurse interaction is under examined from a social perspective. Current research focuses mostly on specific contexts of care delivery and experience related to medical condition or illness, or to nurses’ speciality. Consequentially, this paper is about the social meanings and understandings at play within situated patient-nurse interaction in the community practice setting in a transforming healthcare service.

**Methods:**

Grounded theory methodology was used and the research process was characterised by principles of theoretical sensitivity and constant comparative analysis. The field of study was four health centres in the community. The participants were patients and nurses representative of those attending or working in the health centres and meeting there by scheduled appointment. Data collection methods were observations, informal interviews and semi-structured interviews.

**Results:**

Key properties of *‘Being a good patient, being a good nurse’, ‘Institutional experiences’* and *‘Expectations about healthcare’* were associated with the construction of a category entitled ‘Experience’. Those key properties captured that in an evolving healthcare environment individuals continually re-constructed their reality of being a patient or nurse as they endeavoured to perform appropriately; articulation of past and present healthcare experiences was important in that process. *Modus operandi* in role as patient was influenced by past experiences in healthcare and by those in non-healthcare institutions in terms of engagement and involvement (or not) in interaction. Patients’ expectations about interaction in healthcare included some uncertainly as they strived to make sense of the changing roles and expertise of nurses and, differentiating between the roles and expertise of nurses and doctors.

**Conclusions:**

The importance of social meanings and understandings in patient-nurse interaction is not fully apparent to nurses, but important in the patient experience. Seeking understanding from a social perspective makes a contribution to enhancing knowledge about patient-nurse interaction with subsequent impact on practice, in particular the development of the patient-nurse relationship. The implications are that the meanings and understandings patients and nurses generate from experiences beyond and within their situated interaction are pivotal to the development of their relationship in the transforming community healthcare environment.

## Background

The interaction between patients and nurse is fundamental in their experience of receiving or delivering care [1-5]. Communication skills in interaction are firmly established as requisite to developing the patient-nurse relationship with therapeutic qualities [3,5,6]. The establishment of a therapeutic patient-nurse relationship is argued to be fundamental to quality of care in all health care delivery settings [3,7,8].

Also, the interaction between patient and nurse is linked to patient satisfaction with and the success of healthcare provision – especially in the instance of nurse-led consultations such as those that occur in the community [2,7].

Patient-nurse interaction is purposeful in that it is about the presentation of need by the patient and response with care by the nurse. It involves many processes of engagement that draw on individual meanings and understandings and, as an encounter, can be described as a social experience with requisite social meanings and understandings brought to it. Furthermore, interaction is fundamental to the development of the patient-nurse relationship which, from the patient’s perspective, is quintessential in their experience of being a patient [9-12]. In the literature, patient-nurse interaction is most often related to specific contexts of care delivery, such as hospital admission or palliative care [1,13-15]. Because of this, the patient’s illness and/or the nurse’s professional speciality have provided the focus of research attention.

Whilst a focus on specific illness or symptom care is understandable, it means that examining patient-nurse interaction in the community practice setting has been under-examined in comparison [15-20]. Overall there is lack of literature that examines patient-nurse interaction from a sociological perspective and that also captures the patient experience.

In contrast, the doctor-patient relationship or encounters in the community have been subjected to considerable examination from a range of perspectives [21-23]. A current focus of attention in that literature is decision-making in the clinical encounter between doctor and patient with skew towards patient involvement [24-26]. Patient involvement is the pivot of contemporary healthcare policy in which quality of care improvement is emphasised. Enhancing patient experience and care outcomes play a major part in that emphasis. The emergence of policy that has led to significant changes in healthcare structure and process has resulted in shift to the community setting in terms of where, how and by whom care is delivered [27-30]. In these terms, care delivery in the community experienced by patients has undergone considerable transformation. Also, the roles, responsibilities and expertise of the community multi-disciplinary team have been transformed [31,32].

Structural and situated influences in healthcare and nursing are evolving and values are transforming from biomedical to biopsychosocial [33-35]. That transformation includes movement from illness orientation to health that is perceived as an interdependency of physical, mental, and socio-economic factors [15,36,37]. In that transformation process for those engaged in healthcare, some core values around the precepts of receiving or delivering care may alter, whilst others remain the same [24,38,39]. Also in that process challenges to engagement and involvement in interaction may emerge [6,12,40]. Social meanings and understandings relate to cumulative experience and are brought to and interactive within the engagement of patient and nurse as an involved process.

Movement towards the biopsychosocial model of general medical practice can be traced back to the 1970’s. However, some contradictions in understanding prevail for general practitioners (GPs), nurses and patients [33,34]. The ‘rhetoric or reality’ of the biopsychosocial model has been related to the boundaries of work that GPs identify in that the physical dominates their priorities with the psychological elements of care coming second [2,33,34]. Nevertheless, adoption of the biopsychosocial model has led to a restructuring of the nature of the GP-patient consultation towards therapeutic interaction [40,41]. The therapeutic interaction between patient and GP is now more likely to be subject to negotiation between them [34,41].

Understanding these transformations and the effects of them on the way healthcare is delivered and received in the community is important to understanding the context in which patients and nurses interact.

### Aim

Social meanings and understandings and the influence of them in patient-nurse interaction are concentrated upon in this paper. The key research question was:

What social meanings and understandings can be identified in patient-nurse interaction in the community practice setting and, what influence do they have within that interaction?

### The field of study

The aim of the study reflects traditional concerns with the relationship between individuals in the context in which they interact purposefully. In this instance the context was the community practice setting of four health centres. Health centres were selected because they are where patients and nurses interact purposefully to address health and/or illness needs. The four health centres offer the same level of services in the community with the exceptions of mental health and learning disability. None of these client groups or related nursing staff was included in the participant sample.

Data was collected from the four health centres concurrently and analysed together within the constant comparative analysis process.

The health centres that comprised the field of study are located in areas with geographic and social differences. Taken together, they are representative of the health status of communities across Scotland (Table 1). Population structure; educational attainment, household income; unemployment rate and health indicators are annotated in relation to national averages (Scotland). Population structure relates to the demographic of age; higher meaning more older people than average and lower meaning more younger people. Health indicators relate to significant epidemiological factors that are prioritised in health policy including cardio-vascular disease, respiratory disease, mental health, stroke and cancer.

**Table 1:** Summary of the field of study

**Study area**	**W**	**X**	**Y**	**Z**
Population structure	↔	↓	↑	↔
Educational attainment	↑	↓	↓	↓
Household income	↑	↔	↓	↔
Unemployment rate	↓	↑	↑	↓
Health indicators	↑	↑	↓	↓

↔ Of national average.

↓ Lower than national average.

↑ Higher than national average.

(Extracted from The Scottish Public Health Observatory, health and wellbeing profiles online).

### Ethics

NHS Ethics of Research Committee approval was sought and granted. The study complied with requirements of the Economic and Social Research Council Research Ethics Framework. Having been given written and verbal explanation, participants gave informed consent. The requirements of the Data Protection Act (1998) were complied with fully.

### Recruitment

Participation was sought from adult patients and nurses encountered within the health centres. The participants were considered to be typical of those attending or working in a health centre and meeting by scheduled appointment. The rationale for this approach was that the research was located in the natural setting, the health centre, and the participants were ‘performing’ and interacting in that environment.

### Methodology

Grounded theory methodology was used in the tradition of Glaser and Strauss [42] using the research procedures they specified. Grounded theory was selected for two main reasons. First, it was originally developed to respond to sociologically derived questions and to theorise about social processes. Second, it is a tested methodology in naturalistic inquiry in areas of study that are under investigated such as this [42-45].

## Methods

**Figure 1:**
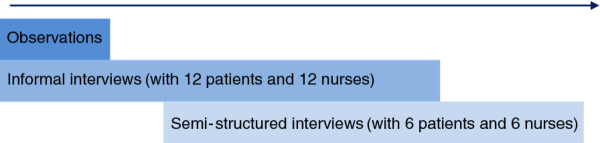
Direction of progression of analysis over time.

Observations, informal interviews and semi-structured interviews that are recognised as core research methods in grounded theory methodology were used (Figure 1). These methods are interactive in grounded theory study in that each informs the other and thus analysis. Data collection took place over a nine month period of time.

The *ad libitum* observations served to provide the contextual basis for the analysis. The interviews were in depth, recorded and transcribed. In a process of constant comparative analysis, data was coded and progressively distilled. Also in that process, lines of inquiry were pursued iteratively with interview participants as conceptual constructs emerged from coding of data. Biases occur in using these qualitative methods in relation to the subjective interaction of the researcher in data collection and analysis and in the potential for observational bias [43,45]. Observational biases were minimised by strategies such as sitting out of view and adopting a non-engaged posture. Biases were addressed primarily by recognising the role they play throughout the research process and by discussion with colleagues. Also, continual engagement with the research aim and questions served to ground data collection and analysis by providing the essential points of reference [44,46].

### Observations

Observations involved watching patient and nurse as they interacted purposefully – the patient receiving care and the nurse delivering. Displays of social and conversational conventions overlain with specific, individual strategies by patients and nurses were observed and subsequently annotated. By providing the contextual basis of the analysis, the annotated observations informed the development of the informal and semi-structured interview schedules.

### Informal interviews

Informal interviews with twelve patients and twelve nurses pursued lines of enquiry related to participants’ background, healthcare experience and interaction. The latter included as examples, forming reactions and developing rapport in their recent interaction. The interviews with patients mostly took place immediately following observation. The nurses were interviewed either on conclusion of the day’s events or the following day.

### Semi-structured interviews

**Figure 2:**
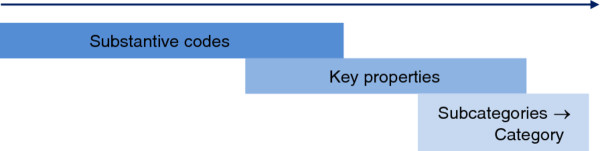
Direction of progression of theory construction.

The key properties emerging in constant analysis of observations and informal interviews formed the basis of the semi structured interview schedules. Six patients and six nurses were interviewed thus and contributed to the enhancement of explanatory power and category development (Figure 2).

### Analysis

When no more variation occurred in the concepts identified from the collected, organised and analysed data theoretical saturation was achieved (within the parameters of this small study). The approach to analysis was consistent with principles of theoretical sensitivity [46] and emergence [47].

Within a constant comparative process, codes were identified from patterns of conceptual meanings in the data and condensed into key properties. Subcategories were generated from a further distillation of those properties. In this way, two categories ‘Experience’ and ‘Investment’ were generated and substantive theory constructed. Achieving theoretical saturation meant that the extent of the explanatory and conceptual power of the analysis could be explained in relation to ‘work, fit and relevance’ in relation to the aim of the research [42].

Robustness was established by: summarising the key points of each interview to each participant and making any amendments they suggested; continually revisiting the data; and referring to field notenotes in the context of each participant’s account. The size of the research field or the participant sample did not constrain the research design or the depth of analysis achieved in relation to meeting the research aim. Mindful of the interactive role of the researcher within constant comparative analysis, refection was deployed purposefully throughout the research process – including discussion with colleagues.

The work of several traditional theorists was useful in the process of analysis. The work of Strauss [48] contributed helpful concepts related to understanding *modus operandi* in interaction in healthcare. ‘The social construction of reality’ as conceived by Berger and Luckmann [49] was helpful in understanding the diverse meanings and understandings that patients and nurses brought to interaction.

Finally, the work of Garfinkel [50] regarding social performance was useful in examining processes of navigation in interaction.

## Results

Findings with integrated commentary derived from the category of ‘Experience’ follow; specifically, the key properties of: *‘Being a good patient, being a good nurse’*, *‘Institutional experiences’* and *‘Expectations about healthcare’*. The participants’ own terms are used throughout and quotes reflect similarities and differences in their views. Consistent with writing grounded theory, literature is integrated throughout.

### Being a good patient, being a good nurse

Being a ‘good’ patient or nurse emerged from data around performance in healthcare. Whilst participants were reflective about role, they recognised the impact of change and brought that recognition to the role they adopted. They also brought those reflections to the prospect of current relationship development and balance within that relationship.

Participants themselves used the terms ‘good patient’ or ‘good nurse’ and had varying views about what that might mean in healthcare today.

Based on their past experience, two patients considered ‘complying’ with care as an important part of managing their social identity as a ‘good’ patient. They described that in interaction patients and nurses played roles, each facilitating the other in achieving an acceptable balance of compliance and control respectively:

"Patient (P) 9: You don’t ask them … they tell you what’s best. You’re in their hands… I’ve no doubt they discuss you and sort it out between them [practice nurse and GP] it’s important to be a good patient for them."

As counterpoint to this, nurse participants expected the patient to be involved in their care to some extent. The involvement nurses expected from patients ranged from asking questions and seeking information, to engagement in decision-making. The role of patient was an expression of belief systems related to experience of interaction learned in the past, and expressed in the present:

"P4: I was brought up to follow what they told you. You just let them get on with it … they were the experts and of course you wouldn’t dream of saying anything unless you were asked…"

Viewing a patient as participatory means that they take responsibility for their own health and wellbeing whilst living their everyday lives within and out with the context of interaction in the health centre. However, a central question arises: how much do individuals as patients delegate their everyday responsibility and how much do nurses assume it in patient-nurse interaction?

The patient has ‘work to do’ in terms of taking responsibility for their illness, and health professionals may conceptualise this responsibility as acting appropriately [51]. Acting appropriately relates to who will do what in respect of patient participation in their own care.

Nurse participants acknowledged understandings from an earlier period in time about how to be a ‘good’ patient held by some older people. Those understandings underpinned the extent to which older patients may or may not participate in their own care. Nurses identified that patients continuing in that mode of understanding created an imbalance of expectations in the patient-nurse relationship. Nurses felt that the impact of such imbalance was in terms of reduced communication and collaboration in patient nurse interaction. In addition for some older patients, contemporary definitions of participation in healthcare have not permeated fully and thus are not fully realised.

It was evident that the adoption of a less active role as a patient continued to be embraced by ten patients and six nurses. Some patients preferred a less active role in their care, which was accepted to a lesser extent by nurses:

"Nurse (N) 16: I know how things have changed. The older generations were brought up to respect authority and do as they were asked with few questions. Some patients are still like that today… despite best efforts"

"N2: It’s quite frustrating really because on one hand you want them to do well, but you do want them to be involved at the same time… you can be too good…"

The feeling of agreement of ten patient participants was ‘leave it to the experts’ the patient should have the option of avoiding involvement in their care in relation to decision-making. ‘Leaving it to the expert’ should be perceived as an alternative mode of action. Nurse participants thought that if a patient is not a ‘good’ one within the parameters of their professional beliefs, the prospect of developing a therapeutic relationship would be affected. Important to resolving this potential conflict is establishing what kind of helping/caring relationship patients and nurses value.

The feeling of agreement that the patient should be active to some extent in interaction with the nurse was mostly expressed by patients who were younger or by those who had had sustained contact with community health services over the years:

"P7: I suppose the whole way of it has changed when you think back over the years… it just happens as time passes. I don’t think you’re aware of it at the time… it’s like everything else… washing machines and colour telly and computers"

Nurse participants also mentioned their experiences of change in a range of ways, including references to the professional and, to a lesser extent, personal:

"N2: I can remember when I first started [nursing] there were so many illnesses they could do nothing about… they didn’t understand them. Think about all those operations for stomach ulcers! We used to keep people in bed for days and convalescence was such a drawn out process. Nursing was such a slog."

"N11: I remember going into hospital when I was child and my parents weren’t even allowed to stay with me. Hospitals were formal places with strict rules and doctors were gods. When I qualified the approach was very much a pathological one… and nursing used a bio-medical approach."

Two older nurses provided these examples and their reflections characterised considerable changes in healthcare and nursing in the United Kingdom in the last thirty years. Various references were made to changes in nursing itself. These comments related to the professional careers of nurse participants in terms of changing roles and emerging opportunities. N11 and N2 discussed how these issues had been influential in their approach to nursing and patient care and provided different perspectives, principally about how the patient had been regarded:

"N11: I think it has mattered a great deal to me how things have changed. It is important that things have moved on. There is much better understanding of who the patient is rather than just what’s up with them… and only looking at problems."

"N2: When people talk about the good old days they forget just how bad things could be… things like not knowing what was going on or being asked… you certainly were not a client… and as a nurse, the same was true half the time."

Despite the assertions by these nurse participants that things have changed in a range of ways, it could be that they were promoting the ideals or ‘received wisdom’ about contemporary practice and policy, rather than accurately describing the impacts of role change in healthcare in particular what might constitute a good patient or a good nurse.

In terms of today’s emphasis upon involvement, the issue of who defines roles in healthcare interaction is at the heart of how the reality of being a patient or nurse is constructed.

### Institutional experiences

Consistent with grounded theory methodology, unexpected findings emerge. In this instance, meanings and understandings drawn on from beyond NHS settings were found to be important to some patient participants and influential in their performance in healthcare settings. It may be the case that patients, who have felt relatively powerless in other institutions by being unable to exert any control over process, may bring those ‘apprehended’ behaviours to their performance in healthcare [48; 50; 51]. That may mean that their belief in engaging in involvement is reduced. That may mean they are perceived as less interactive by nurses. Four patient participants in particular were observed to await instruction from the nurse; they did not assume it was their turn rather waited until the nurse formally invited them to follow and to then to be seated and, continued to respond to instructions without initiating action themselves for example, rolling a sleeve up.

The four patients referred specifically to experiences they had had in other institutions including the Department of Social Security (DSS) and Social Work Department (SWD).

These specific references were made during interviews and emerged around the issue of knowing what to do as *modus operandi* when meeting the nurse and in need-care interaction. The patients described the rituals they felt obliged to follow if they were to achieve the purpose of their visit to either DSS or SWD:

"P11: It’s like take a number … like at the counter in Tesco … wait your turn, go where you’re telt [told] … dae what you’re telt … an away."

"P13: You have to just go through the hoops or you’ll get nowhere…they’ve got a way of working … you fit in … get the business done, that’s it"

Participants believed that certain behaviours were appropriate in similar settings, including DSS and the Health Centre. Whilst they identified that systems related to basic organisation were essential, for example to avoid queue jumping, they felt a sense of resignation about going through pre-determined hoops in that what you did and how you did it was not open to negotiation. The participants expressed difficulties with this in a range of ways. In particular, whilst they disliked *‘being a number’* and having to *‘go along with it’*, they were resigned to it as appropriate behaviour to adopt to achieve their purpose. Four participants identified that it is reasonable to be asked to *‘sit down there’* or *‘go there’*, especially if you were a novice in such a place as the DSS. However, the key issue seemed to be that you would probably be left to pick up clues from others and copy what others did:

"P17: I didn’t have a clue what to do… couldn’t really make head nor tail of it. Eventually I could see some were waiting to have forms checked, some were being called… eventually I worked out that folk at one side were waiting to make inquiries like me… so I moved and sat at the end…"

P17 describes clearly the process of working out what to do in an unfamiliar setting as did others. In order to navigate their way through the unfamiliar, they drew on wider experiences, a process that served them only to a limited extent. The key issue that perplexed them was the lack of signposts that they expected to be there by custom and practice. These signposts were not only the physical, for example, lack of inquiry signs, but also lack of opportunity to interact face to face. The participants noted that there often seemed to be a lack of social engagement with fellow clients and staff in DSS and/or SWD. In effect, the lack of information and supportive actions for example, effective signposts or human communication, hindered individuals’ opportunities to problem solve. The alternative and available action was to be passive and compliant in a process over which they felt they had little control because clues to aid *modus operandi* were largely concealed. The understandings generated contributed to participants’ beliefs about their competence in institutional settings and the control that could be exerted in settings that they identified as similar, such as the health centre.

Beliefs about parameters of control were related to the individual’s experiences and beliefs about how to navigate in an institutional setting. These beliefs were influenced by interpretations of how to behave conventionally, *‘going through the hoops’* as the participant put it. Berger and Luckmann [49] propose that individuals navigate in the social world by identifying and typifying recognisable patterns of activity. By this process, individuals can predict how their activities will be evaluated and reacted to by others. Berger and Luckmann argue that: ‘The social reality of everyday life is thus apprehended in a continuum of typifications, which are progressively anonymous as they are removed from the ‘here and now’, of the ‘face-to-face situation’. Although the health centre environment was identified as more facilitative than other contexts in terms of interaction, how individuals have ‘apprehended’ interaction in other contexts may be transferred and contribute to the complexity of performance in patient-nurse interaction as exemplified by P13: 

"P13: These places have their way of working … the same way more or less… you just settle yourself down to it and let them get on with it. Aye, best way"

The experiences identified by patient participants who had had considerable experience in other institutions (DSS and SWD) support this argument.

Patient participants described that at times their sense of self was undermined. One patient participant used the term ‘diminished’ in relation to being a number [P11] *‘like at the counter in Tesco’*.

The sense of being undermined or diminished was particularly true of those who described the encounters significant to them out with the health centre. The significance of these encounters was both the link between the reason for the visit, for example seeking welfare/benefits, and the effect the adherent navigation processes had on them. The demands and challenges of organisational structures and processes compromised their sense of self-determination, including in interaction in healthcare and thus for patient-nurse relationship development.

### Expectations about healthcare

Participants’ expectations related to healthcare were sometimes those of uncertainty and they brought that to their performance in situated interaction. They connected their experiences in healthcare in the past to experiences in the present day as an interdependency linked to their performance. In doing so they referred to changes in healthcare, nursing and the roles of patients and nurses.

The inclusion of psychosocial aspects in assessment was the prevalent feature included by nurses in their conceptualisation of delivering nursing care. Their conceptualisation related well to patients’ identification of nursing as being more than giving physical care. Whilst four nurse participants expanded on issues of development and change, as shown in previous examples, the points they raised were also identified to some extent by the other nurses.

These views suggest that the approach to caring by nurses may be different from that expected by patients. Those patients are likely to be older and familiar with traditional bio-medical models of care.

In the terms of the nurses cited whose approach to care went beyond bio-medical matters, a tension between their approach and patients’ understanding of nursing and healthcare emerges:

"P11: I haven’t seen the doctor for years… I didn’t even realise they had practice nurses… took me a while to understand what her drift was [role and approach]. I suppose I thought the doc would give me some tablets and keep an eye on it [hypertension] … but she [the nurse] asks me all sorts about lifestyle and all that. She does more than just check it like I thought she would… she takes her time… very good"

In need-care interactions, nurses’ interpretation of nursing ‘the whole person’ commonly prevailed over patients with expectations of a bio-medical approach to caring. The accomplishment of nurse participants seemed to be to win patients over to their approach to caring, rather than it being explained and agreement established. In other words, patients generally ‘caught the drift’ through co-operating in patient-nurse interactions as P11 did. Importantly, ‘really’ being listened to was identified as critical in the consultation process as eight patient identified explicitly:

"P4: She’s a genuine lassie… puts up with my stories and moans and groans… she doesn’t make me feel like a silly old fool"

"P8: We work things out… even if it’s a daft thing. I like the way they remember you… and relax you really"

As in these examples, it was important to patients to feel that they could express themselves without pressure and in comfort, and nurses welcomed that. Interestingly, the two patients just cited [P4 and P8] identified that it is important that, in addition to listening, nurses also accepted that patients needed to tell their story without feeling ‘silly’ or ‘daft’. In terms of confidence and trust, this related to patients’ concerns about preservation of integrity and the importance placed upon behaving appropriately and being accepted:

"N9: above all, patients just need to feel that they can just talk; it’s about them … how things get sorted out, wee things and feelings get picked up"

The motivator to overcoming any uncertainly was the development through interaction of the patient-nurse relationship (which was valued highly where it existed). Nurse participants recognised that a patient’s emphasis upon the ‘expert’ ‘knowing best’ created an imbalance in their relationship.

Participants revealed how they created and sustained meanings and understandings of healthcare and nursing by talking about their changing expectations and beliefs and relating them to the present day. Meanings emerge in local contexts and situations through interaction with others, and wider historical understandings are drawn upon to do so [48,49,52]. The emergence of meanings is an achievement in everyday life in which meanings are created and sustained in performance in interaction [50].

For the participants, creating and sustaining meanings and understandings emerged in, and of, interaction as it took place. In the process of creating meaning within interaction, patients and nurses draw upon the past to perform in the present – an evolutionary process in which the past articulates with the present.

In this process, some past meanings are sustained and new meanings are created about how to perform in interaction. Their expectations and beliefs have or are changing as a consequence of their contemporary experiences:

"P9: It’s daft when you think about it… only going to the health centre when you’re ill… it’s no called the illness centre. Years ago health didn’t come into it much at all… as they say; the future of the community depends on health. At least now, things are more open… like mental health. Nurses have much more to do now… they’re trained for it … they work alongside other people like the doctor rather than separate."

Comments such as these represented a largely positive view of changes in community healthcare, and identify what were seen as positive aspects of the evolving role of the nurse and transformation in healthcare:

"N12: I know that some people attending the health centre are quite baffled by who does what now until they get used to it. A lot of that relates to the extended roles we [nurses] have, and the specialist roles that are designed to meet patient’ needs … although I don’t think they always realise that. I suppose it has become very different over the last few years"

Patients have a range of views regarding expertise in the health centre, including confusion about what expertise is and with whom it lies.

These views were explicitly echoed by five patient participants whilst others were won over by the competence of practice nurses:

"P6: I wasn’t sure at first… I was used to the doctor monitoring me. But I got used to the nurse and she knows how to adjust things and we sort them out [blood glucose levels]"

"P13: It’s just that you’re used to something else… the lady doctor always sent for you and checked you over and did the [cervical] smear. The nurse does it now and it’s just the same… no bother. She checks the rest of you out as well so it’s time well spent on yourself eh!"

Some patients stated that they would rather see a doctor in circumstances where a medical condition was subject to regular review, for example, diabetes mellitus:

"P2: I just have more confidence … I prefer medical advice and expertise"

"P7: I don’t doubt her proficiency in lots of ways… I’m used to dealing with the doctor … I just believe the doctor is the best person in my circumstances"

Views such as these do not necessarily express an adherence by patients to a reductionist bio-medical approach, or to hierarchical notions that the services of the doctor are superior to those of nurses. Rather, these views may represent a sense of security generated by familiarity with the doctor as provider of care. In these terms, transformations in health care may result in a sense of insecurity related to expertise for some [53].

Nurse participants did not include the same meanings identified by patient participants in their accounts; however, they did include reference to changes in nursing including their professional contribution:

"N11: before you were the nurse that worked along with the GPs in your district. The patients were his and you assisted with skills with patients with lots of different needs… I would say it was more about tasks though, not nursing skills as we know it today."

"N9: Nowadays we have our own caseloads and take responsibility for managing the patients… it’s a turnaround in the way we practice as nurses working as specialists with our multi-disciplinary colleagues and we do bring lots of experience and training to it [the role]"

The nurses’ layers of experience had accrued over time and were expressed in the style of their professional practice and interaction. Those layers included the encounters and experiences they had had in diverse healthcare settings, especially in acute hospitals.

Changing working practices related to interaction include the idea that a ‘blurring’ process occurs as roles evolve [32,35,54]. The blurring process is in terms of who does what in a division of ‘caring’ labour as the role and expertise of the nurse extends. The blurring of roles has implications for the patient in terms of understanding interaction with individuals with a range of professional expertise in the health centre.

Roles and professional identities have been redefined in the community practice setting with a movement towards person centred care [16,33]. Expertise has been redistributed with the ‘up skilling’ of nurses leading to more complex patient cases being seen by the GP as s/he determines. GPs and some nurses are higher up the complexity hierarchy which is bio-medically focused. In this way a ‘hierarchy of appropriateness’ emerges for the patient [33]. Redistribution of roles and expertise contradicts person centredness in that presenting with a biomedical issue becomes the determining factor as to whether you see a doctor or a nurse as a patient in the health centre [3,34]. For some patients this may mean they may not be able to consult with the healthcare professional of their choice and/or preference. From interviews with patients, it is evident that these arrangements for managing care delivery have not been driven fully by them. Also, it is evident that these arrangements may not be apparent to or easily understood by patients.

## Discussion

Important social meanings and understandings are brought to patient-nurse interaction in the community practice setting and impact upon performance. Performance includes the preferred role as patient or as nurse linked to expectations about interaction in the community practice setting. Those meanings and understandings have implications for how we understand performance in interaction and relate it to the development of the patient-nurse relationship.

Some evidence emerged that patients differentiate between their relationship with the doctor and their relationship with the nurse. Within that differentiation, perceptions of expertise and power are embedded. In their endeavours to engage in healthcare, some patients understood that nurses and doctors had different and discreet roles to play in healthcare. In these terms, it may be the case that the notion of professional hierarchy remains pervasive for patients. It is the case that medical encounters and work [3,9,33,35] and patient-nurse encounters and interaction [1-5] have been subject to some examination. However, the perspective of patients is under examined in relation to understanding and acceptance of their role [12,18,19]. As contributing evidence, patients and nurses in this study experienced some uncertainty in striving to make sense of the challenges of the changing health service, including roles and expectations. Transformations at the coalface of healthcare and nursing are also somewhat ambiguous for some patients and nurses.

Ambiguity emerges from perceptions that decisions concerning treatment and care should be embedded in the traditional domain of the doctor, rather than the nurse. Others qualified their beliefs about this and located the doctor’s power in the realm of medical diagnosis and treatment in a multi-disciplinary team, and referral to other experts, including nurses. Of note is that uncertainty created challenges in constructing a performance as patient in particular interfaced with who was ‘expert’.

Those challenges of engagement are more important in patient-nurse interaction than previously recognised and suggest that whilst models of medical and nursing care have evolved including roles and expertise [2,15,34], the public may not have grasped the changes and all that they encompass. However, if the movement to biopsychosocial and therapeutic models of care is more ‘rhetoric than reality’ [34] then the expectations of those patients seeking a biomedical approach to care will be met.

The articulation of past and present experiences in healthcare was more than the impact of the passing of time or nostalgia. It was about constructing the reality of being a patient or nurse today. Nurses’ reference to changes in nursing matched the meanings and understandings of patients in terms of articulation of past and present experiences. That articulation is a new thread of understanding about making sense of transforming healthcare by and thus for patients and nurses.

The emergence of *modus operandi* adds to the existing literature [22-25] by capturing the wider institutional experiences that may be brought to patient-nurse interaction by patients. In relation to *modus operandi*, the complexity of patient-nurse interaction should include consideration of how individuals experience interaction in a range of institutions, because they bring meanings and understandings from that experience to their performance in healthcare. The experiences of patient participants beyond healthcare settings were not entirely positive and were related to difficulties in processes of navigation.

Participants described that they had difficulty in identifying recognisable patterns of activity that they could engage with. Difficulties in navigation resulted in a loss of sense of control. Participation and involvement in patient-nurse interaction were jeopardised for those who had experienced feelings of being powerless in other institutions. Subsequent implications for the patient-nurse relationship can be surmised.

Taking into account that this was a small study, several areas emerged in analysis that deserve further study specifically, *modus operandi* in healthcare, the import of interaction experienced in other institutions and, the differentiation of the roles of patient, nurse and doctor.

## Conclusions

The centrality of social meanings and understanding in patient-nurse interaction is not fully apparent to nurses, but important in the patient experience and to how they perform in role. Seeking understanding from this social perspective makes a contribution to enhancing knowledge about patient-nurse interaction with subsequent impact on practice, in particular the development of the patient nurse relationship.

Organisational structures and processes may compromise the self-determination of individuals by imposing formalities, routines and rituals that appeared to be mostly impenetrable in terms of navigation. The inclusion of these meanings is important to understanding the generation and construction of meanings in situated interaction, especially by the patient in a healthcare setting. Consideration of those more complex social meanings expands understanding of patient-nurse interaction and thus is very relevant in healthcare that is transforming.

Individual experiences of health and social services are influential in perceptions of roles, performance and *modus operandi*. Whilst health and social services are very relevant to this argument, further understanding could lie in experiences in other social institutions, for example, education, religion and judiciary related (probation and/or prison). For patients and nurses this could also mean other health or social care providers, such as private healthcare or care homes. How individuals have experienced and understood interaction in other institutions contributes to understanding the complexity of situated patient-nurse interaction: an insight missing in existing knowledge.

## Competing interests

The author declares that she has no competing interests.

## Pre-publication history

The pre-publication history for this paper can be accessed here:

http://www.biomedcentral.com/1472-6955/11/14/prepub
